# Homology analysis of malware based on ensemble learning and multifeatures

**DOI:** 10.1371/journal.pone.0211373

**Published:** 2019-08-26

**Authors:** Di Xue, Jingmei Li, Weifei Wu, Qiao Tian, JiaXiang Wang

**Affiliations:** College of Computer Science and Technology, Harbin Engineering University, Harbin, Heilongjiang, China; Ulm University, GERMANY

## Abstract

With the exponential increase in malware, homology analysis has become a hot research topic in the malware detection field. This paper proposes MHAS, a malware homology analysis system based on ensemble learning and multifeatures. MHAS generates grayscale images from malware binary files and then uses the opcode tool IDA Pro to extract opcode sequences and system call graphs. Thus, RGB images and M-images are generated on the image matrix. Then, MHAS uses convolutional neural networks (CNNs) as base learners to perform bagging ensemble learning to learn features from the grayscale images, RGB images and M-images. Next, MHAS integrates the nine base learners using voting, learning and selective ensemble (in that order) and maps the integration results to the result matrix. Finally, the result matrix is again integrated using the learning method to obtain the final malware classification result. To verify the accuracy of MHAS, we performed a malware family classification experiment, that included samples of 10 malware families. The results showed that MHAS can reach an accuracy rate of 99.17%, meaning that it can effectively analyze and identify malware families.

## Introduction

Malware is the common name for software that is not intended to be executed by a user, has a malicious purpose, and completes a malicious function. A wide variety of malware exists, including Trojans, viruses, worms, DDoS, zombies, backdoors, malicious builds, adware, ransomware, and so on [1]. Currently, given the popularity and widespread use of the Internet, people are increasingly dependent on software, which is a prime motivator behind the growth and rapid spread of malware. Human malware inspectors can find thousands of malware instances every day, but the emergence of various automated tools has shown that the speed with which malware mutates on the Internet is far faster than people realized. For example, Kaspersky Labs detected 15,714,700 malicious objects [2] in 2017, while the number of malicious files detected by McAfee Labs increased to 79 million per day in 2018 Q1 (Q1 means the first quartal), up from 45 million in 2017 Q4 (Q4 means the fourth quartal) [3]. At the same time, as the attacks become more advanced and continuous (e.g., APT [4], advanced persistent threat), the definition of malware has become increasingly broad, malware attack scenarios have grown increasingly complex, and the means of transmission have become increasingly obscure (e.g., malware can now propagate via Bluetooth [5]). To avoid detection, malware authors adopts techniques such as polymorphism, deformation and other methods. However, studies have found that most unknown malware is derived from known malware, and some malware repeatedly use certain functions or code libraries. Thus, malware evolved from a common ancestor through code reuse or whose behavior is similar have successor relationships and are called “homologous”. Therefore, finding the homology among samples plays an important role in tracing attack sources, restoring operating environments, and preventing attacks.

In recent years, to move beyond traditional malware detection methods, an increasing number of researchers have proposed using machine learning methods to solve large-scale malware detection problems by searching for items such as API use [6] and specific function calls [7] through static, or dynamic analysis [8]. However, most of these methods extract only one malware feature; consequently, it is impossible to comprehensively analyze the homology of malware. Therefore, problems that involve extracting more features while managing feature-extraction costs and extracting more useful information from original malware samples will be the main work of researchers in the malware field. The goals are to achieve more complete model automation and to improve the accuracy and efficiency of malware homology analysis.

This paper presents a malware homology analysis system (MHAS) based on ensemble learning and multifeatures. MHAS falls into the static analysis category, and it can extract multiple features: grayscale images from the original malware data, opcode sequences transformed into RGB images, and system call graphs transformed into M-images. An M-image is a special type of grayscale image. Compared with a grayscale image containing 256 grayscale values, an M-image has only M grayscale values. For example, when M = 2, the image has only two grayscale values (0 for white and 255 for black).

This paper contains three main parts. First, MHAS uses a convolutional neural network (CNN) as a base learner that learns three types of feature views; this CNN acquires the outline features, instruction sequence characteristics, and control structure features of malware. Then, MHAS proposes a method based on ensemble learning to integrate the ensemble results of the base learner; this approach is called ensemble learning reintegration (ELR). The ELR includes both the ensemble strategy of the base learner and the ensemble strategy applied to the ensemble strategy of the base learner. The ELR further improves the accuracy of malware classification. Finally, we conduct a series of experiments with MHAS, to investigate its true position rate and accuracy rate for malware family classification by varying the number of features and the integration strategy. Experiments show that MHAS achieves a better classification performance than do DNN or CNN approaches that handle only single malware features.

The remainder of the paper is organized as follows. Section 2 describes the key research content and research results in the field of malware detection. Section 3 explains the process for generating the M-images that represent malware system call graphs as well as the generation of grayscale and RGB images. Section 4, we construct the ensemble strategy of the base learner and the ELR (ensemble learning reintegration) based on the idea of bagging ensemble learning. The experimental results and analysis are presented in Section 5. Finally, Section 6 summarizes this paper and proposes future work.

## Related work

Malware detection has always been a hot topic in the computer security field. Detection plays an important role in finding homology among the samples to trace the attack source, restore operating environments, and prevent attacks. Because most homologous malware instances stem from the same author or the same team, they often have highly similar software structures. Malware analysis relies on static analysis methods based on call graphs [9] or sequence-based methods [10] and dynamic analysis methods based on dynamic taint propagation [11]. As background to the method proposed in this paper, in this section we describe previous malware classification related work from three perspectives: image processing, machine learning and deep learning.

### Malware analysis based on image processing

Nataraj et al. [12] was the first to propose visualizing malware binary files as grayscale images and using similarity calculations between images to classify malware families. The authors performed experiments-on 9,458 samples representing 25 malware families and reached a classification accuracy of 98%. This method was also able to recover obfuscated encryption technology. A number of researchers extended the method proposed by Nataraj [13–15] using multiple classification methods, including machine learning models, to test multiple malware corpora containing more than 100,000 malware samples. The results of these methods all indicate that image texture analysis method (which belongs to the static analysis category) can achieve results similar to dynamic analysis. In response to the problem of the size of the grayscale images extracted by Nataraj, Han KS et al. [16] proposed a new malware family classification method to determine malware variants similarity that first converted malware binary files into grayscale images and then applied a histogram similarity measurement method to compare the similarities of grayscale image entropy maps. Experiments showed that this method can achieve a relatively high classification accuracy. HAN et al. [17] proposed a method of feature extraction and detection of malicious code based on texture fingerprints. First, the malicious code is mapped to an uncompressed grayscale picture, which is segmented into blocks by the texture segmentation algorithm. Then, the texture features in each block are extracted by the grayscale cooccurrence matrix algorithm to establish a texture fingerprint index structure. Finally, a weighted, synthetic, multisegmented texture fingerprint similarity matching method is used to detect malicious code variants and unknown malicious code. When applied to the analysis and detection of six malicious code families, this method’s highest accuracy reached 85.77%.

In addition to generating grayscale images, malware classification based on image technology has several other types of image applications. For example, Han KS et al. [18] proposed a malware visualization method based on combining static and dynamic analysis in which an RGB image is first generated from opcode sequences extracted from malware samples; then, a key block is selected to extract opcode sequences using a method that dynamically executes malware. The method calculates image similarity using pixel color information from the RGB image. A method for extracting a representative image of a malware family was also proposed to reduce the number of comparisons required for classifying unknown samples. The accuracy of this malware classification approach can reach 98.96%. Tingting Wang et al. [19] proposed a new visualization method to address the problem of small training sets. First, the opcode sequence extracted from the malware binary file was converted into a color image. That image is then normalized by histograms, dilated and eroded. Principal component analysis is applied to extract features and enhance the images. Finally, a Support Vector Machine (SVM) classifier based on RGF kernel functions was proposed to classify the malware. This approach achieved high detection accuracy from a limited training set.

### Malware analysis based on CNNs

CNNs are widely used in deep learning. Due to the presence of the perceived field in the CNN, the CNN has good locality and great potential for graph similarity measurements. Malware classification methods based on CNNs [20–22], have achieved varying results. Tobiyama et al. [20] applied deep learning to malware classification. First, the API call sequence was recorded as process behavior, and a feature extractor was built using a long short-term memory (LSTM) language model. Then, the feature is extracted from a trained recurrent neural network (RNN) and feature images are generated. Finally, feature images annotated with malware or benign labels were assembled to form the input to the CNN. A total of 81 malware log files and 69 benign software log files were used for training, and the system classified 26 types of malware from 11 families. The accuracy of this method reached 96%. Kolosnjaji B et al. [21] used a neural network with convolutional layers and circular layers to classify malware using system call sequences as a feature. In a dataset consisting of 4,753 malware samples, this combined neural network architecture achieved an accuracy of 85.6% and a recall of 89.4%. To solve the algorithmic complexity of the subgraph isomorphism problem, ZHAO et al. [22] proposed a structure that used a CNN to process API call graphs of malware code. From a dataset that included eight malware families and 200 malwares instances, the accuracy of this method reached 96.7%.

### Malware analysis based on multiple features

Most of the existing work in the field of malware classification based on image technology is dealing with a single feature, such as the use of binary files, opcode sequences, and API call sequences. These features are insufficient to cover all the features of malware. So, for this issue, some researchers proposed classification methods that integrates multiple features. Liu L et al. [23] proposed a malware analysis system based on machine learning, which consists of three modules: data processing, decision making, and malware detection. The features they extract include grayscale images, opcode sequences, and import functions. Then these features were input into the decision-making. Finally, the malware was classified using the shared nearest neighbor (SNN) clustering algorithm. The system classified more than 20,000 malware, and the classification accuracy reached 98.9%. Aziz Makandar et al. [24] proposed a multi-classification method of malware based on SVM from the perspective of image processing. This method constructed a 56-dimensional texture feature vector using multiple features such as Gabor wavelet, GIST, and discrete wavelet transform. And this method selected 8 malware families on the Maling data set to classify, included 1,610 samples in the training set and 1,710 samples in the testing set, and the classification accuracy reached 98.88%. Huang et al. [25] proposed MtNet, which uses multi-task learning and a deep neural network (DNN). The extracted features include API 3-grams, which are three consecutive API call sequences, and API call parameters. 50,000 features were extracted using mutual information, and then 4,000 features were extracted using the random projection (RP) for dimension reduction. The training set and the test set used by them are 4,500,000 and 2,000,000, and they have the largest data size currently used in the research field of malware detection. The binary error rate and multiple classification error rate were 0.358% and 2.94%, respectively.

The related works presented above show that using image structures to represent malware information can better preserve the integrity of the malware. Malware classification methods using multifeatures and deep learning have achieved good results. However, as malware increases exponentially, malware classification methods face continual challenges. To meet these challenges, MHAS first extracts many useful features from large amounts of raw data and converts the feature information into image formats, thereby preserving the integrity of the malware. Then, features are learned from the images through the automatic feature-learning characteristics of the CNN. Finally, the multiple CNN classification results are integrated to obtain further classification accuracy.

## Feature extraction

### MHAS overview

**Fig 1:**
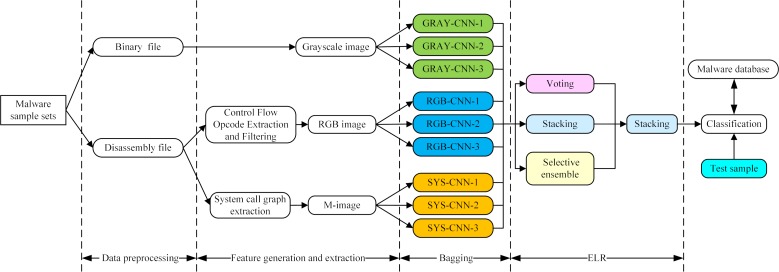
Overview of MHAS.

This paper proposes MHAS, which is based on ensemble learning and multifeatures. As shown in Fig 1, the system uses grayscale images, RGB image matrices and M-images as feature views, and uses CNNs as the base learners for the bagging ensemble learning process. MHAS chooses the voting method, learning method and selective integration as the ensemble strategy applied to the base learners. Finally, it adopts the learning method to integrate the ensemble strategy of the base learners for the second time.

MHAS’s malware homology analysis, process is divided into four phases.

1) Data preprocessing: Malware sample data is preprocessed to convert an executable file to be analyzed into two preprocessed files. One is a binary file, and the other is a disassembly file, which is generated by the disassembly tool IDA Pro.

2) Feature generation and extraction: The original binary stream can embody the random outline features of malware; the control flow opcodes embody the instruction sequence characteristics of the malware; and the system calls map the control flow executed inside the malware. Thus, the binary stream file, control flow opcode and system calls extracted as malware features fully exploit the malware information. However, to generate a data format appropriate for input to the base learner, CNN, in subsequent bagging ensemble learning, the binary stream file and the disassembly file must be it is processed again. In this step, MHAS generates grayscale images from the binary stream file, and extracts the control flow opcodes and system call graphs from the disassembled file to generate RGB images and M-images, respectively.

3) Bagging: In the feature generation and extraction phase, 3 feature views are generated as input. This stage uses CNNs, which have achieved excellent performance in the image classification field, as the base learners.

4) ELR: When the malware sample data is input, MHAS first adopts 3 different ensemble strategies, forming 9 types of basic learner results for the 3 types of feature views. Then, it uses the learning method to integrate the results of the basic learners’ ensemble strategies to obtain the final classification result.

This section explains the generation and extraction of the grayscale images, RGB images, and M-images. The construction of GRAY-CNN-X, RGB-CNN-X, and SYS-CNN-X (X = 1,2,3) and the bagging ensemble learning are described in Section 4.

### Grayscale image generation

Many similar units occur in images that repeat and have regular distributions; these are called texture features. Texture features describe the spatial distribution and spatial interrelation between the gray levels of images. Texture features are also global features that describe the nature of the surface structure in the image. Because a CNN weakens local bias and possesses rotation invariance, it has great advantages in the field of image recognition. In malware analysis, the malware binary file is converted to a grayscale image. Images belonging to the same malware family have greater similarity in their overall layouts and texture features. Moreover, the computer-generated grayscale image is not affected by image resolution and the environment. Malware classification based on image texture features is a novel method that has been demonstrated to be an effective static analysis tool [12].

**Fig 2:**
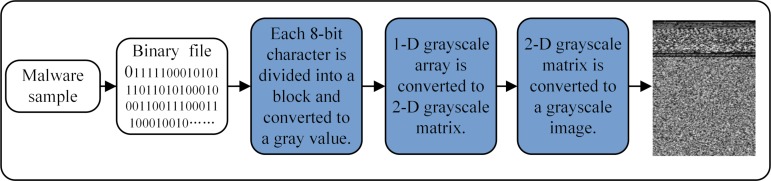
Grayscale image conversion process.

Fig 2 shows the process of converting malware into grayscale images and using image processing techniques to visualize and classify malware. Processing the original Windows executable file and converting it into JPG images requires the following steps: 1) The executable file is treated as an original binary stream, forming the input data. 2) Binary stream file conversion: The binary stream sequence is split into many small blocks, each consisting of 8 characters. Then the blocks are converted to image pixels with 256-level gray values. The conversion scheme maps byte values from 0 (black) to 255 (white). 3) Grayscale image generation: A one-dimensional grayscale array is converted into a two-dimensional grayscale matrix in an orderly manner. The width and height of the two-dimensional grayscale matrix are determined based on the size of the malicious code file [12], to obtain an uncompressed JPG grayscale image.

### RGB image generation

To extract the opcode sequence from the malware sample data, MHAS first uses IDA Pro to decompile the executable file and generate a file in .asm format. IDA Pro is a powerful disassembly tool that effectively addresses Intel x86 assembly instructions in the Windows executables [26]. The .asm file consists of a series of opcodes divided into operators and operands. The operators are determined by IDA Pro's predefined Intel x86 instruction list, and the operands include variables, values, and addresses. The frequency, position and sequence of the operators in the code reflect not only the programming style of the software programmer but also the functional characteristics of the software. Therefore, most studies extract only operators as the opcode sequence [27].

#### Control flow opcode sequence extraction

Some researchers in the field of malware analysis use opcode sequences for analysis. For example, Santos I et al. [28] used a fixed-length n-gram opcode sequence. Although this approach is simple to construct, it can also lose information concerning the software features. Han et al. [16] constructed a variable-length n-gram opcode sequence using the jump instructions (e.g., "jmp", "jz", etc.) in x86 opcodes. Although this approach preserves certain syntax information, it ignores the malware control flow information. MHAS applies a control-stream-based opcode sequence extraction method to disassembled malware opcode sequences. The extracted subsequence not only retains the malware control flow information, but also fully utilizes the behavioral information in the operation code sequence. Subsequences have unique entry and exits points. Thus, each subsequence includes a complete sequence flow with no other relationships. However, jumps, calls, parallel executions or sequential flows may occur among subsequences. The MHAS subsequence construction algorithm is described as follows:

**Table:** 

**Algorithm 1.** MHAS **s**ubsequence construction algorithm.	
**Input:** Disassemble file *file*	
**Output:** Control Flow Opcode Sequences Array *SUB*	
1:	*address*←the lowest address of the code area of *file*; *A*[0..*n*-1]←*address* array of split points; flag←false;
2:	**while** !flag **do**
3:	*statement*←statement of the *address*;
4:	**if** *statement* = the first statement of the paragraph or target statements of conditional transfer statements or unconditional transfer statements or the next statement of the jump statements **then**
5:	*A*[*i*]←*address*;
6:	flag←false;
7:	Update code area *address* by address increment;
8:	**else if** *address* ≠ the last statement in the *file* code area **then**
9:	flag←false;
10:	Update code area *address* by address increment;
11:	**else** flag←true;
12:	**end while**
13:	**for** *j*←0 to *n*-1 **do**
14:	*subsequence*←statements between two split points *A*[*j*] and *A*[*j*+1];
15:	phrase array *B*[0..*l*-1]←split *subsequences* with whitespace;
16:	**for** *k*←0 to *l*-1 **do**
17:	**if** *B*[*k*] = Intel x86 assembly instruction set **then**
18:	opcode array *O*[0..*m*-1]←*B*[*k*];
19:	**else** delete *B*[*k*];
20:	**end for**
21:	*string*←Stitch the opcode in array *O* into a string in order;
22:	control flow opcode sequences array *SUB*[0..*s*-1]←*string*;
23:	**end for**
24:	**return** *SUB*

**Fig 3:**
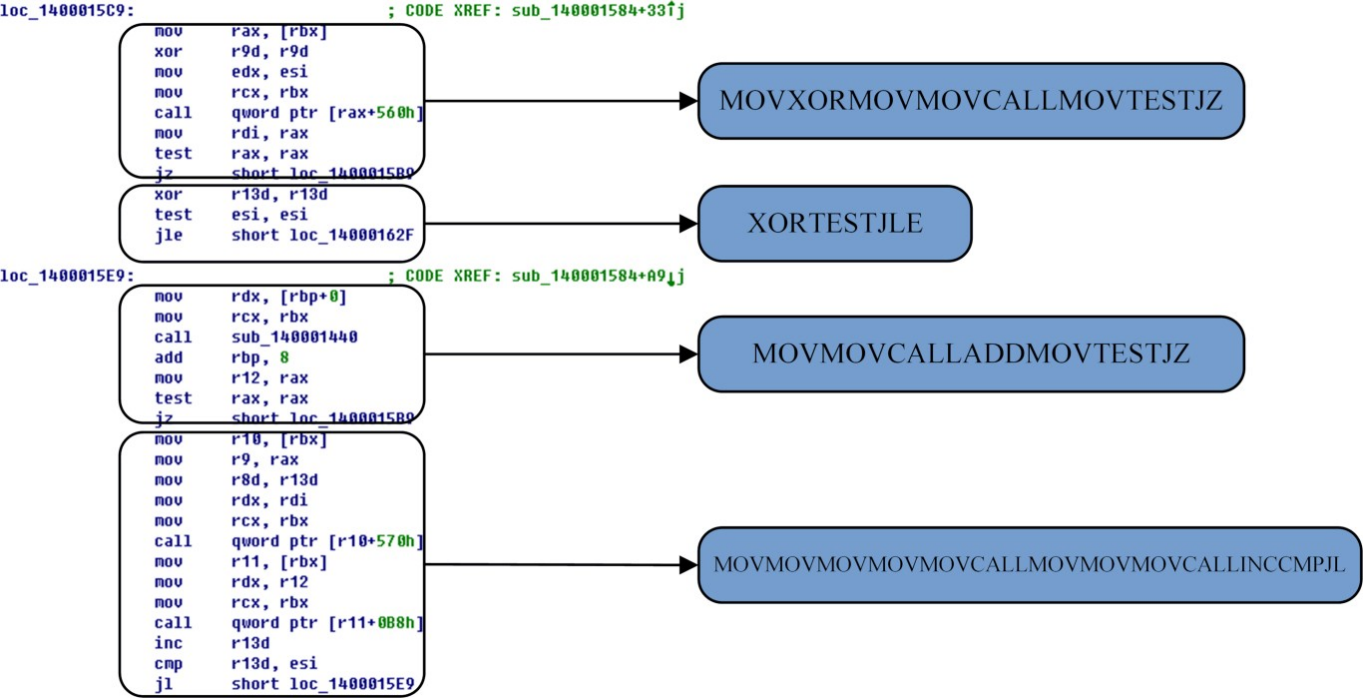
Partition of opcode sequences.

In the static analysis technique, the disassembled instruction sequence does not have a statement that the control flow cannot reach; therefore, Algorithm 1 can traverse all the codes. The x86 assembly instructions "jmp", "jz", "ja", "ret" and "retn", and others are either unconditional or conditional execution transfer statements whose next statement is a split point. The "call" statement is used as to execute a call; control flow returns after the call completes. Therefore, this statement can be treated as an ordinary sequential flow. For example, Fig 3 shows a subsequence division diagram of the disassembled malware in which the first division point is the first instruction because it contains the address referenced in the virtual address “140001584+33”. The second division point is the instruction "xor r13d, r13d" because it is the instruction following the conditional jump instruction "jz short loc_140001589". By analogy, the subsequence partitioning method in Fig 3 can extract four opcode sequences:"MOVXORMOVMOVCALLMOVTESTJZ","XORTESTJLE","MOVMOVCALLADDMOVTESTJ" and "MOVMOVMOVMOVMOVCALLMOVMOVMOVCALLINCCMPJL".

The opcode sequences used by MHAS do not target all malware disassembly results subsequences because using all subsequences for the analysis also extracts some nonmeaningful opcode sequences, which not only increases the difficulty of distinguishing the malware features, but also increases analysts’ workloads and increases the time required to generate RGB images. The frequency of each subsequence in the malware sample data is counted, and the feature is filtered based on the subsequence frequencies. The subsequence whose frequency falls into a defined interval is selected as the software feature. When the frequency value exceeds the upper bound or falls below lower bound of the threshold, the sequence will not be used as a software feature. This approach not only degrades the features into traditional software feature codes but also reduces unnecessary interference to the machine learning algorithms caused by some common code.

#### RGB image pixel generation

In addition to converting malware raw data directly into grayscale images, it is also possible to convert the assembly files obtained by decompiling malware into images. To accomplish this, Han KS et al. [18] proposed a method for extracting the opcode sequence in a malware .asm file and convert it into a hash value to generate an RGB image matrix. For the opcode sequence generated in Section 3.3.1, MHAS uses the SimHash function [29] to convert it to a 40-bit fingerprint and divide it into five 8-bit characters. Each character represents pixel coordinates and colors of the opcode sequences in the RGB image. SimHash is a locality sensitive hashing (LSH) method. Its main idea is that mapping two adjacent data points in a high-dimensional data space into a low-dimensional data space increases the probability of data adjacency. Two data items that were not originally adjacent will also have a high probability of being non-adjacent in the low-dimensional space. Therefore, when the opcode sequences are similar, the output hash values are also similar and they are mapped onto similar coordinates in the image matrix.

**Fig 4:**
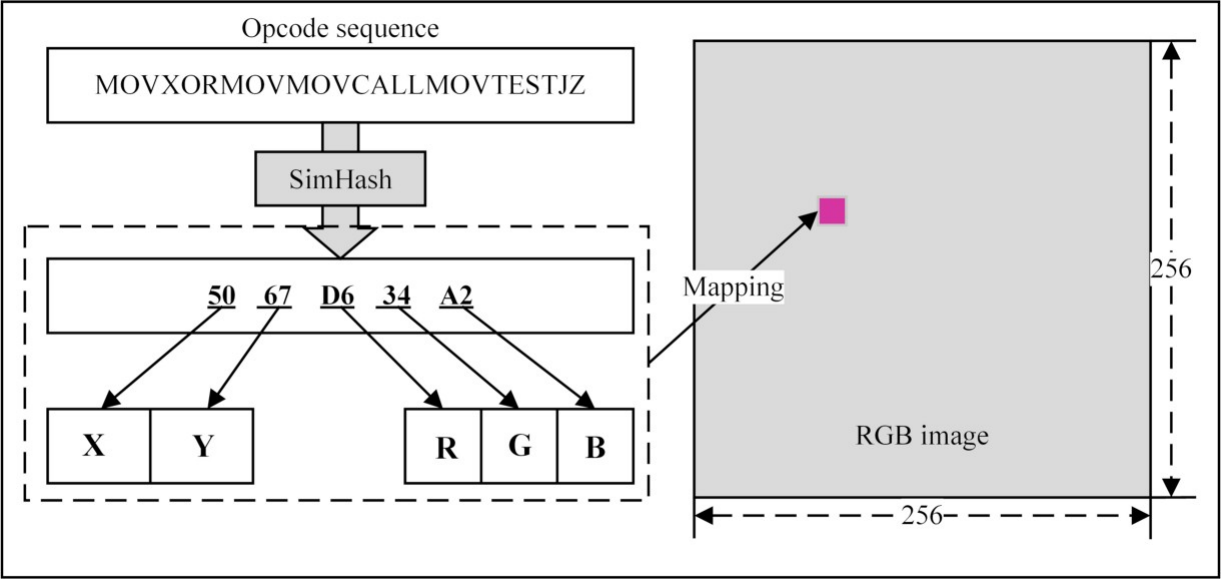
Generation of pixel points using opcode sequences.

Fig 4 shows the process of converting the opcode sequence "MOVXORMOVMOVCALLMOVTESTJZ" into the pixel coordinates and colors in the RGB image. The hash value of the 40-bit fingerprint determines the pixel's X-Y coordinate and RGB color. In the disassembled file, the number of opcodes is definitely smaller than the file size, and the opcode sequence extraction further reduces the number of items to be processed. Thus, compared to the size of the grayscale image in section 3.2, the size of RGB image matrix is 256×256. This approach effectively reduces the collision probability of the SimHash function. When the pixels are mapped as shown in Fig 4, when they overlap because their coordinates are the same, the sum of the RGB colors forms a new pixel color, and the summation result can exceed 255 (0xFF). For example, the SimHash value of the opcode sequence m is 5067D634A2H, and the SimHash value of the opcode sequence n is 5067346425H. Consequently, the new, mapped pixel’s X-Y coordinate is (50, 67), and its RGB color is (FF, 98, C7). Finally, the extracted opcode sequences are mapped individually to a 256×256 RGB image, thereby forming a malware feature image that is used as the feature input to the base classifier.

### M-image generation

Malware needs to use various services provided by the Windows system to perform malicious actions. For example, opening a file, terminating a process, modifying a registry entry, etc., all require interaction with the Windows system. This interaction is accomplished through calls to the Windows system. Therefore, to identify malicious behaviors, it is important to track the order of system calls during program execution. Different malware families have different goals, and malware can be classified based on the differences in execution goals. Relative to the instruction sequence and function call graph, the system call graph has a higher level of abstraction, and reduces the data volume. Moreover, it is not affected by code obfuscation techniques because it omits the software code details.

#### Related definitions

Definition 1: System Call Graph

A system call graph G is a directed graph consisting of three elements, G = (V, E, W), where V is a finite set of vertices *V* = {*v*_1_,*v*_2_,…,*v*_*n*_}, and each vertex corresponds to a system call function; *E* = {(*v*_*i*_,*v*_*j*_)|*v*_*i*_,*v*_*j*_∈*V*} is a directed edge set in which an edge between vertex *v*_*i*_ and vertex *v*_*j*_ represents a system call from *v*_*i*_ to *v*_*j*_, not in the opposite direction. W is the weight of the directed edge E, where *w*_*ij*_ represents the number of times that vertex *v*_*i*_ invoked vertex *v*_*j*_; *w*_*ij*_ and *w*_*ji*_ are two separate weights. The system call graph G indicates the execution sequence between system calls and, consequently, the overall structure of the target program.

Definition 2: System Call Matrix

A system call matrix A is an n-order matrix, where elements *a*_*ij*_ in matrix A represent the edge weight of the system call from *v*_*i*_ to *v*_*j*_.

$$a_{ij}=\{\begin{array}{ll} w_{ij} & \exists (v_{i},v_{j})\\ 0 & \neg (v_{i},v_{j}) \end{array}$$

Definition 3: Vertex Degree

G is a directed graph. The sum of the edge weights whose endpoint is vertex *v*_*i*_ in G is called the in-degree of *v*_*i*_, recorded as *ID*(*v*_*i*_). The sum of the weights of the edge whose starting point is vertex *v*_*i*_ in G is called the out-degree of *v*_*i*_, recorded as *OD*(*v*_*i*_).

#### System call graph Extraction

To obtain the system call function, we first use IDA Pro to decompile the executable file and generate the .asm file. Then, we scan the entire assembly code file and select the statements containing call and jump instructions, such as jnz and jmp. The functions called by the call instruction fall into two categories: custom functions and import table functions. If the calling target is a custom function, the scan enters the custom function, scans its internal assembly statements, and filters its internal system call functions. After the filtering is completed, the different functions are connected based on the execution order of the system call functions and the jump structure of the jump instructions. Finally, the overall system call graph G of the malware is established.

To perform malware family clustering, the extracted system call graph should reflect the common characteristics and minimize the unique characteristics among the malware families. Therefore, we need to extract the key subgraph G′ from the overall system call graph G. This subgraph, G′, reflects not only the commonalities within a given family but also the differences among different families. The vertices of the key subgraph G′ should be composed of important system call functions. MHAS uses the PageRank algorithm to calculate the degree of importance of each vertex in the system call graph G. Then, it classifies the system call function and selects the L functions with the highest degree of importance to form the vertices of the key subgraph G′. Formula (1) shows how the PageRank algorithm [30] calculates the score:

$$PageRank(p_{i})=\frac{1-q}{SUM}+q\sum_{p_{j}}\frac{PageRank(p_{j})}{L(p_{j})}$$ (1)

Whereby, *p*_*i*_ and *p*_*j*_ are the pages of the studied malware, *PageRank*(*p*_*i*_) is that page's PageRank, *SUM* is the total number of pages, *q* is the damping coefficient, which is the probability that the user will arrive at a page and continue to browse backwards at any time, and *L*(*p*_*j*_) is the number of output pages, *p*_*j*_.

Because the user cannot randomly access the vertices in the system call graph, Formula (1) needs to be modified by deleting the damping coefficient *q*. Considering that a system call function is a fixed type of function, the frequency with which a function appears in the malware is also an important aspect of malware behavior. This paper analyzes the malware family *S* = {*S*_1_,*S*_2_,…,*S*_*R*_} (where *R* is the number of malware families) downloaded from the VX Heaven platform. For example, for the malware family *S*_1_, we count the frequency of each system call function appearing in the family sample and calculate the term frequency-inverse document frequency (TF-IDF) of the system call function, calculated as follows:

$$TF‐IDF=\frac{n_{i}}{\sum_{k}n_{k}}•\log\left(\frac{D}{d_{i}+1}\right)$$ (2)

Whereby, *n*_*i*_ is the number of times the system call function *i* appears in the malware family sample, $\sum_{k}n_{k}$ is the sum of the occurrences of all system call functions in *D*, *D* is the number of samples of the malware family, and *d*_*i*_ is the number of samples containing the system call function *i*.

According to the objectives that MHAS needs to achieve, the calculation formula (2) of TF-IDF is introduced into Formula (1) to reflect the importance degree of each vertex, modifying Formula (1) as follows:

$$VRank(v_{i})=\frac{TF‐IDF_{i}}{SUM}+TF‐IDF_{i}•\sum_{v_{j}}\frac{VRank(v_{j})}{L(v_{j})}$$ (3)

Whereby, *VRank*(*v*_*i*_) is the importance degree of the vertex *v*_*i*_, *SUM* is the number of malware samples included in the family *S*_*j*_, and *L*(*v*_*j*_) is the number of vertex *v*_*j*_ calls to other vertices, that is, the out-degree of *v*_*j*_, recorded as *OD*(*v*_*j*_). By recursively calculating Formula (3), the importance degree of the vertex is obtained when the result is stable.

**Fig 5:**
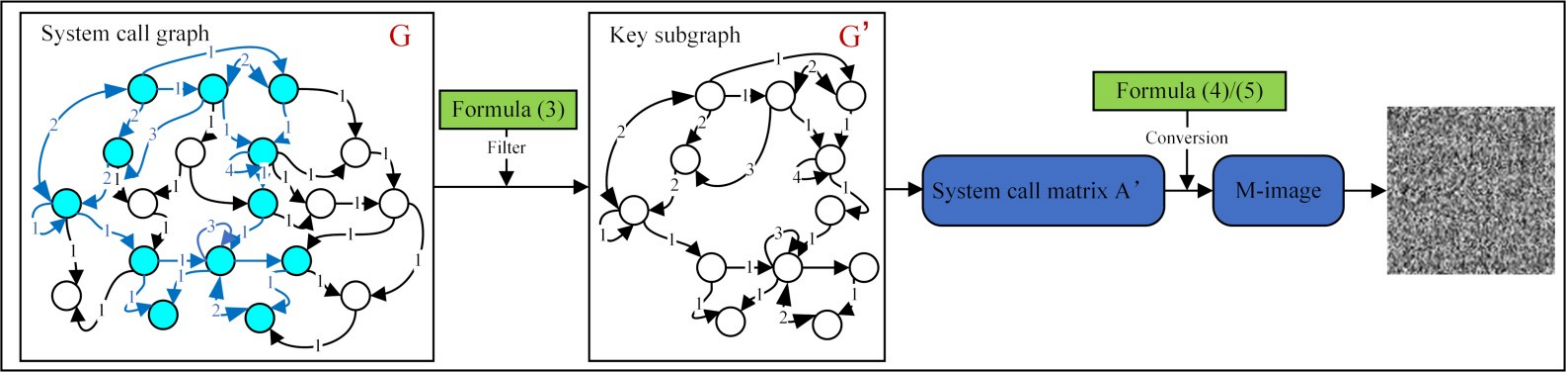
Processing flow of the system call subgraph.

According to the importance of the vertices, we select the most important L vertices, that is, the L most important system call functions in the malware family *S*_*j*_. For each sample in the malware family *S*_*j*_, we traverse the entire assembly file to find and filter out the L important system call functions, and then connect the L vertices according to the system call matrix A to form the key subgraphs G′. When the CNN processes an input image, it is convolved using pixels as the basic unit; therefore, the key subgraph G′ needs to be processed so that it can match the CNN input. The specific processing flow of the system call subgraph is shown in Fig 5.

Let the key subgraph of the system call graph be G′, which contains the L important vertices *G*' = {*g*_1_,*g*_2_,..,*g*_*L*_|*g*_1_,*g*_2_,…,*g*_*L*_∈*V*}. The constructed system call matrix A′ is an L×L matrix. The elements *a*_*ij*_ in matrix A′ represent the weights of the system call vertices from *v*_*i*_ to *v*_*i*_. Then, we transform matrix A′ into an M-image. To make the pixel values in the M-image individually correspond to the elements in matrix A′, the key subgraphs of each sample of the malware family are traversed and the largest element max(*a*_*ij*_) in the system call matrix A′ is found. The relation between *M* and max(*a*_*ij*_) is as follows:

$$M=\left\{ \begin{array}{ll} 2 & \max(a_{ij})=1\\ \max(a_{ij})+2 & \max(a_{ij})\neq 1\;and\;odd\;number\\ \max(a_{ij})+1 & \max(a_{ij})\neq 1\;and\;even\;number \end{array}\right.$$ (4)

After determining the value of *M*, the elements in the system call matrix A′ need to be mapped into the M-image. The mapping relation is as follows:

$$gray‐value_{ij}=\{\begin{array}{ll} 0 & a_{ij}=0\\ \frac{a_{ij}}{M-1}•256-1 & a_{ij}\neq 0 \end{array}$$ (5)

Whereby, *gray-value*_*ij*_ is the value of the pixel in the *i-th* row and the *j-th* column in the M-image, and *a*_*ij*_ is the element in the system call matrix A′.

After the above steps have been completed, the key subgraph of the system call graph of the malware has been changed to an L×L M-image. The M-image serves as the input of the SYS-CNN-X.

## Ensemble learning system

After MHAS generates grayscale images from malware (the RGB images representing the opcode sequences and the M-images representing the system call graphs), we need to construct nine basic learners and four ensemble strategies to learn and analyze these feature views. Because CNNs achieve good effects in the image classification field, the three feature views extracted by MHAS are eventually converted into image forms. Therefore, for MHAS, we chose to use CNNs as the base learner. However, a CNN is a type of learner that is susceptible to sample disturbances; thus MHAS adopts the bagging ensemble learning algorithm with bootstrap sampling [31], which trains the base learners by compensating for the CNNs susceptibility to sample disturbances.

### Base learner construction

The MHAS uses the three extracted feature views as CNN inputs, and it improves the accuracy of malware classification by utilizing the advantages of CNN's translation invariance and by sharing weights to reduce the number of network free parameters. The MHAS constructs three CNN network structures: GRAY-CNN-X, RGB-CNN-X, and SYS-CNN-X. The configuration parameters are shown in Table 1. The output size format in Table 1 is [*num*, (*row*, *col*)], where *num* is the number of feature maps and *row*×*col* is the feature map size.

**Table 1:** Parameter list of the three CNNs.

Network layer type	Size	Output size^g^	Output size^r^	Output size^s^
Input layer*	-	[1,(r, c)]	[3,(256, 256)]	[1,(L, L)]
Convolutional Layer*	8 3×3 Convolution kernel	[8,(r, c)]	[8,(256, 256)]	[8,(L, L)]
Max pooling layer*	2×2,stride 2	[8,(r/2,c/2)]	[8,(128, 128)]	[8,(L/2,L/2)]
Dropout layer*	-	[8, (r/2,c/2)]	[8,(128, 128)]	[8, (L/2,L/2)]
Convolutional Layer*	16 3×3 Convolution kernel	[16, (r/2,c/2)]	[16,(128,128)]	[16, (L/2,L/2)]
Max pooling layer*	2×2,stride 2	[16, (r/4,c/4)]	[16,(64, 64)]	[16, (L/4,L/4)]
Dropout layer*	-	[16, (r/4,c/4)]	[16,(64, 64)]	[16, (L/4,L/4)]
Convolutional Layer*	32 3×3 Convolution kernel	[32, (r/4,c/4)]	[32,(64, 64)]	[32, (L/4,L/4)]
Convolutional Layer*	32 3×3 Convolution kernel	[32, (r/4,c/4)]	[32,(64, 64)]	[32, (L/4,L/4)]
Max pooling layer*	2×2,stride 2	[32, (r/8,c/8)]	[32,(32, 32)]	[32, (L/8,L/8)]
Dropout layer*	-	[32, (r/8,c/8)]	[32,(32, 32)]	[32, (L/8,L/8)]
Convolutional Layer*	64 3×3 Convolution kernel	[64, (r/8,c/8)]	[64,(32, 32)]	[64, (L/8,L/8)]
Convolutional Layer*	64 3×3 Convolution kernel	[64, (r/8,c/8)]	[64,(32, 32)]	[64, (L/8,L/8)]
Max pooling layer*	2×2,stride 2	[64, (r/16,c/16)]	[64,(16,16)]	[64, (L/16,L/16)]
Dropout layer*	-	[64, (r/16,c/16)]	[64,(16,16)]	[64, (L/16,L/16)]
Convolutional Layer*	64 3×3 Convolution kernel	[64, (r/16,c/16)]	[64,(16,16)]	[64, (L/16,L/16)]
Convolutional Layer*	64 3×3 Convolution kernel	[64, (r/16,c/16)]	[64,(16,16)]	[64, (L/16,L/16)]
SPP layer**	3-layer pyramid	1344-D vector	-	-
Max pooling layer***	2×2,stride 2	-	[64,(8,8)]	[64, (L/32,L/32)]
Dropout layer*	-	1344-D vector	[64,(8,8)]	[64, (L/32,L/32)]
Fully connected layer*	512 Neurons	[512, (1,1)]	[512, (1,1)]	[512, (1,1)]
Fully connected layer*	512 Neurons	[512, (1,1)]	[512, (1,1)]	[512, (1,1)]
Fully connected layer*	512 Neurons	[512, (1,1)]	[512, (1,1)]	[512, (1,1)]
Soft-max*	R classification	[R, (1,1)]	[R, (1,1)]	[R, (1,1)]

* These layers are common among GRAY-CNN-X, RGB-CNN-X and SYS-CNN-X (X = 1,2,3).

** This layer only belongs to GRAY-CNN-X.

*** This layer is common both RGB-CNN-X and SYS-CNN-X.

^g^ This output size belongs to GRAY-CNN-X.

^r^ This output size belongs to RGB-CNN-X.

^s^ This output size belongs to SYS-CNN-X.

MHAS constructs a CNN network structure based on VGGNet [32], which uses smaller convolution filters in deeper parts of the network. GRAY-CNN-X, RGB-CNN-X and SYS-CNN-X in Fig 1 have similar network structures. As shown in Table 1, the CNN has 22 layers (excluding the input layer), including 8 convolutional layers, 5 pooling layers, 5 dropout layers, 3 full-connection layers, and an output layer. All the convolutional layers use a 3×3 convolution kernel with a step size of 1; the number of convolution kernels in the eight layers are 8, 16, 32, 32, 64, 64, 64, and 64. Because the size of the feature map does not change when the feature map passes through a convolutional layer, a 1-pixel edge fill is performed on each input feature map in the convolution layer. The first four pooling layers of the GRAY-CNN-X model and all the pooling layers of the RGB-CNN-X and SYS-CNN-X models use max pooling with a 2×2 sliding window and a step size of 2. Because the last fully-connected layer of the CNN requires that the input feature maps be the same size, the general CNN network structure needs to preprocess the image to unify the image size. However, segmentation reduces the correlation between the blocks, and compression reduces the effective information in the image. In response to this problem, the last pooling layer of the GRAY-CNN-X network proposed by MHAS uses spatial pyramid pooling (SPP) [33] instead of max pooling. The output of the SPP layer is a k×B dimension vector, where B represents the number of bins and k represents the number of filters in the last convolution layer. This fixed-dimensional vector forms the input to the fully-connected layer, allowing the inputs to be images of any size. MHAS uses 3-layer pyramid pooling and obtains vectors of 4×4×k, 2×2×k, and 1×1×k dimensions. Then, the output of SPP is connected to a 21×k dimensional vector and output to the fully connected layer.

To prevent network overfitting, the CNN includes a dropout regularization layer with a probability of 0.5 after each pair of convolutional and pooling layers. Behind the last dropout layer are three fully connected layers with 512 output neurons and one output layer (R-SoftMax classifier). In addition, to enhance the convergence performance of the CNN network, MHAS uses the Leaky ReLU activation function [34], with a uniformly distributed weight initialization and batch normalization [35].

### Ensemble strategy

**Fig 6:**
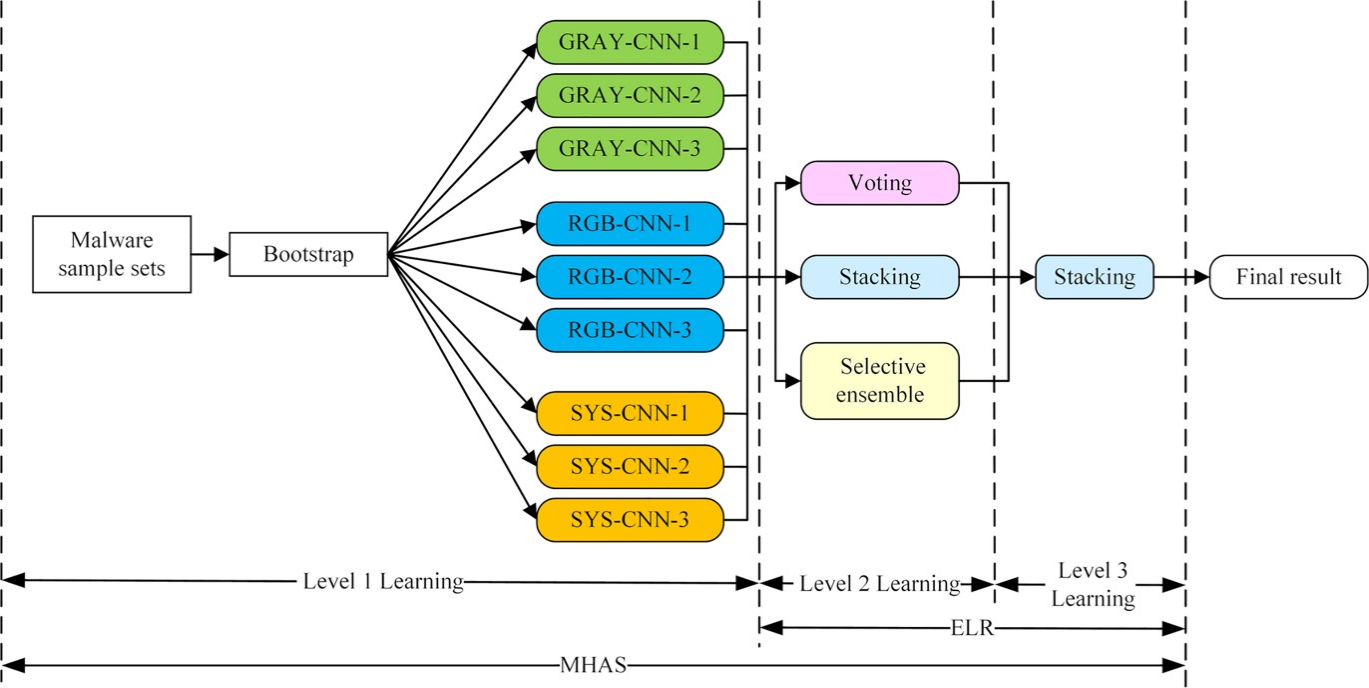
Learning process of the MHAS.

Ensemble learning is used to identify malware by integrating the results of multiple base learners, thereby improving the final accuracy and reducing the false alarm rate. For the grayscale images, RGB images, and M-images processed by MHAS, we propose a method called ensemble learning reintegration, ELR. The MHAS divided into four phases in Fig 1 can also be divided into three levels of learning, as shown in Fig 6. Level 1 learning is the process of training the base learner (bagging). Level 2 learning is the process of integrating the base learner. Level 3 learning is the process of integrating the results of the ensemble strategy form Level 2 learning. Both level 2 and level 3 learning use ensemble learning approaches to form the ELR.

In level 1 learning, three base learners are trained on each feature view. The base learners *h*_*i*_(*i* = 1,2,…,9) predict a marker from the set of class labels {*S*_1_,*S*_2_,…,*S*_*R*_} (where *R* is the number of malware families). We represent the predicted output of *h*_*i*_ in sample *x* as a 1×R result vector *result*_1(*x*|*h*_*i*_),defined as:

$$result\_1(x|h_{i})=\{h_{i}^{1}(x),h_{i}^{2}(x),\ldots ,h_{i}^{R}(x)\}$$

Whereby, $h_{i}^{j}(x)$ denotes the output of the base learner on the category tag *S*_*j*_. Each vector element $h_{i}^{j}(x)$ takes the value 1 (the malware belongs to family *S*_*j*_) or 0 (the malware does not belong to family *S*_*j*_).

In level 2 learning, the three ensemble strategies use absolute majority voting, learning methods, and selective ensemble, which are respectively represented by *e*_*1*_, *e*_*2*_, and *e*_*3*_. MHAS defines the output of each ensemble strategy as a 1 × R result vector, *result*_2(*x*|*e*_*l*_,*l* = 1,2,3):

$$result\_2(x|e_{l})=\{e_{l}^{1}(x),e_{l}^{2}(x),\ldots ,e_{l}^{R}(x)\}$$

Whereby, *result*_2(*x*|*e*_*l*_) represents the predicted result of sample *x* by ensemble strategy *e*_*l*_ and $e_{l}^{j}(x)$ represents the output of ensemble strategy *e*_*l*_ on the category tag, *S*_*j*_. The vector element $e_{l}^{j}(x)$ takes the value 1 (the malware belongs to family *S*_*j*_) or 0 (the malware does not belong to family *S*_*j*_).

For absolute majority voting, if $\sum_{i=1}^{9}h_{i}^{j}(x)=0.5•\sum_{k=1}^{R}\sum_{i=1}^{9}h_{i}^{k}(x)$, we define the formula

$$result\_2(x|e_{1})=\{e_{1}^{1}(x),e_{1}^{2}(x),\ldots ,e_{1}^{R}(x)|e_{1}^{j}(x)=1\cap \sum_{k\neq j}e_{1}^{k}(x)=0\}$$

That is, if a marker receives more than half the votes, the resulting prediction for that marker position $e_{1}^{j}(x)$ is 1, and the remaining positions are 0.

The learning method in level 2 learning uses Stacking [36]. Stacking refers to a basic learner as a primary learner, while the ensemble learner is called the secondary learner. For each malware sample data in MHAS, the nine 1×R result vectors *result*_1(*x*|*h*_*i*_) obtained by the primary learners are combined into a 9×R feature vector, which is used as the input feature for the secondary learner. The final output is a 1×R result vector, *result*_2(*x*|*e*_2_):

$$result\_2(x|e_{2})=\{e_{2}^{1}(x),e_{2}^{2}(x),\ldots ,e_{2}^{R}(x)|e_{2}^{j}(x)=1\cap \sum_{k\neq j}e_{2}^{k}(x)=0\}$$

Both the voting method and the learning method use all the built-in base learners for integration, which increases the required storage space. The MHAS selects the “selective ensemble” strategy during level 2 learning [37]. We evaluate *h*_*i*_(*i* = 1,2,…,9) using the evaluation method, which removes the base learner with little effect and poor performance from the existing base learners and then selects *T* base learners for integration, obtaining a 1×R result vector *result*_2(*x*|*e*_3_):

$$result\_2(x|e_{3})=\{e_{3}^{1}(x),e_{3}^{2}(x),\ldots ,e_{3}^{R}(x)|e_{3}^{j}(x)=1\cap \sum_{k\neq j}e_{3}^{k}(x)=0\}$$

In level 3 learning, we use the learning method to integrate the results *result*_2(*x*|*e*_*l*_) of the level 2 learning. The learning method is expressed with *e*, and we obtain a 1×10 result vector *result*_3(*x*|*e*):

$$result\_3(x|e)=\{e^{1}(x),e^{2}(x),\ldots ,e^{R}(x)\}$$

Where *result*_3(*x*|*e*) represents the prediction for the sample *x* by the ensemble strategy *e* and *e*^*j*^(*x*) represents the output of ensemble strategy *e* on the category tag, *S*_*j*_. The vector element *e*^*j*^(*x*) takes the value 1 (the malware belongs to family *S*_*j*_) or 0 (the malware does not belong to family *S*_*j*_). Based on the results of level 3 learning, MHAS judges which family the malware belongs to and completes the malware detection.

### MHAS algorithm

Algorithm 2 describes the detection process used in MHAS based on the ensemble learning and multifeatures described in this paper. In Step 5, we use 9 CNNs as the base learner to classify, and obtain a bagging ensemble learning model based on multifeatures. Then, in Steps 9 and 12, we propose an ELR method that first integrates the classification results of the base learners and then integrates the integrated results of the base learners by using an integration strategy to obtain a set of classification results. Finally, in Steps 17 to 22, the abnormality of the classification result is processed to ensure the accuracy and fault tolerance of the MHAS model.

**Table:** 

**Algorithm 2.** Classification algorithm of MHAS based on ensemble learning and multifeatures.	
**Input:** Malware sample *file*	
**Output:** Malware family classification result *number*	
1:	Conversion file type: binary file *b_file*←*file*; disassembly file *d_file*←*file*;
2:	Extracting feature views using three feature extraction methods;
3:	*M*[0..8][0..9] is a 9×10 matrix vector; *R*[0..2][0..9] is a 3×10 matrix vector;
4:	**for** *x*←1 to 9 **do**
5:	Using the classification of the feature views on the base learner *h*(*x*), the classification vector *result_1*[0..9] is obtained;
6:	*M*[*x*-1][0..9]←*result_1*[0..9];
7:	**end for**
8:	**for** *y*←1 to 3 **do**
9:	Using the integration of *M*[0..8][0..9] on the integration strategy *e*(*y*), the classification vector *result_2*[0..9] is obtained;
10:	*R*[y-1][0..9]←*result_2*[0..9];
11:	**end for**
12:	*result_3*[0..9]←*R*[0..2][0..9] by integration strategy *e*;
13:	Calculate the sum of the elements in *result_3*[0..9];
14:	**if** sum = 1 **then**
15:	Query the element *result_3*[*l*] = 1 in *result_3*[0..9];
16:	*number*←*l*+1;
17:	**if** sum > 1 **then**
18:	Query the element *result_3*[*l*] = 1 in *result_3*[0..9];
19:	Randomly select one of the elements, *result_3*[*l*];
20:	*number*←*l*+1;
21:	**if** sum = 0 **then**
22:	**return** error;
23:	**return** *number*

## Experiments and analysis

### Experimental preparation

The experimental data set for this paper was collected primarily through the VX Heaven website [38], which contains 270,000 tagged malware samples. MHAS focuses on 32-bit executable files on the Windows platform; we selected 10 malware families with 300 malware samples for the Windows operating system. The number of malware families is denoted as *R* = 10. As shown in Table 2, each malware family contains 30 samples and 10×10-fold cross-validation is performed.

**Table 2:** Malware sample family.

Number	Malware family	Quantity
No.1	Backdoor.Win32.Bifrose	30
No.2	Backdoor.Win32.SdBot	30
No.3	Backdoor.Win32.IRCBot	30
No.4	Trojan-Downloader.Win32.Lemmy	30
No.5	Trojan-Downloader.Win32.IstBar	30
No.6	Trojan-Dropper.Win32.Tab	30
No.7	Trojan-Dropper.Win32.Eva	30
No.8	Virus.Win32.HLLP.Semisoft	30
No.9	Virus.Win32.Gpcode	30
No.10	Worm.Win32.Deborm	30

MHAS converts each malware sample to two file formats: the malware source file (binary file) and the assembly file decompiled by IDA Pro. For each source file, MHAS generates a grayscale image. For each assembly file, MHAS extracts the opcode sequences and the system call graph, and then converts them to an RGB image and an M-image, respectively.

### Experimental design

This paper conducts experiments to investigate 4 aspects. First, we compare the influence of the number of key subgraph vertices (Section 3.4) on the result. Second, we compare the influence of the ensemble strategy choice on the classification result. Third, we compare the influence of feature types and quantity on the result. Fourth, we compare the accuracy of MHAS to the accuracy of other analysis methods to illustrate the advantages of MHAS.

We used the true positive rate to evaluate the MHAS analysis results and accuracy rate to compare MHAS with the results of other methods. Specifically, in MHAS, the number of samples belonging to the malware family S that are correctly predicted as belonging to malware family S is the true positive (TP) rate, while the number of samples not belonging to the malware family S that are erroneously predicted as belonging to malware family S is the false positive (FP) rate. The number of samples not belonging to the malware family S that are correctly predicted as not belonging to the malware family S is true negative (TN) rate, and the number of the samples belonging to the malware family S that are erroneously predicted as not belonging to the malware family S is false negative (FN) rate. The true positive rate and the accuracy rate are defined as follows.

The true positive rate (TPR) is the proportion of samples belonging to the malware family S that are correctly predicted as the malware family S out of all the samples belonging to the malware family S.

$$TPR=\frac{TP}{TP+FN}$$

The accuracy rate (AR) refers to the proportion of correct predictions S out of all the tested samples.

$$AR=\frac{TP+TN}{TP+TN+FP+FN}$$

### Experimental results and analysis

#### The influence of the number of key subgraph vertices on the result

**Fig 7:**
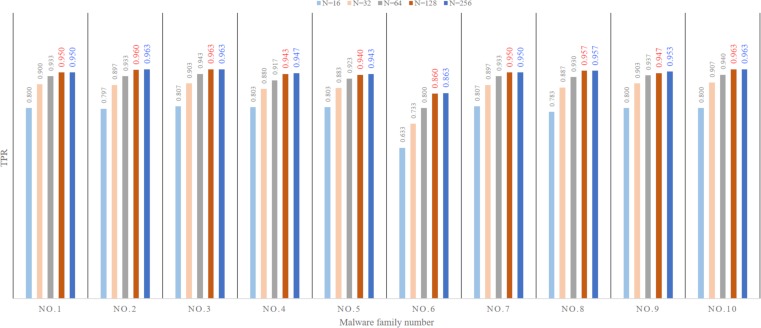
The influence of the number of the key subgraph vertices on the result.

We experimentally compare the influence of the choice of the number of key subgraph vertices L (see Section 3.4) on the classification results. The results are shown in Fig 7. The experimental results are obtained from M-images through three base learners and the ELR. In Fig 7, the ordinate indicates the true positive rate, the abscissa indicates the malware family number, and each line indicates the classification result of different numbers of vertices L. As shown in Fig 7, although the malware classification effect improves as the number of vertices L increases, when the number of vertices *L*≥128, the TPR of each malware family does not change much but the feature extraction time increases. Therefore, M-images are performed using 128 for the number of the key subgraph vertices; that is, the MHAS extracts 128×128 M-images.

#### The influence of ensemble strategies on the result

**Fig 8:**
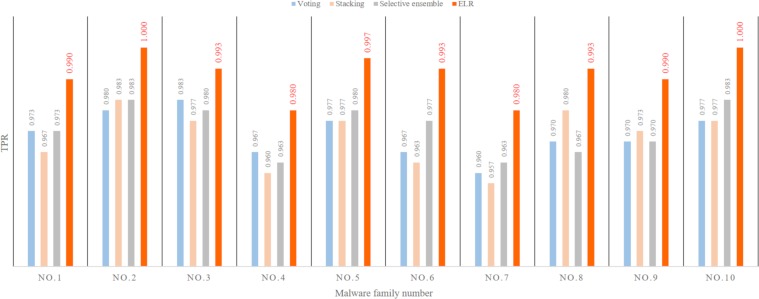
The influence of ensemble strategies on the result (multifeatures).

Ensemble learning is a machine learning method that first trains a series of base learners, and then uses an ensemble strategy to integrate the individual learning results to obtain a better result than that obtainable from a single learner. For the three feature views extracted by MHAS, the integration strategy voting, stacking, selective ensemble and the ELR proposed in this paper are analyzed. The results are shown in Fig 8, in which the ordinate represents the true positive rate and the abscissa represents the malware family number. As Fig 8 shows, the experimental results may not be able to any integration strategy with an absolute advantage for all malware family classifications, but the results when using ELR are significantly better than the results when using the other three integration strategies. The average true positive rate of the ELR represents an increase of 2%~4%.

#### The influence of feature type and quantity on the result

**Fig 9:**
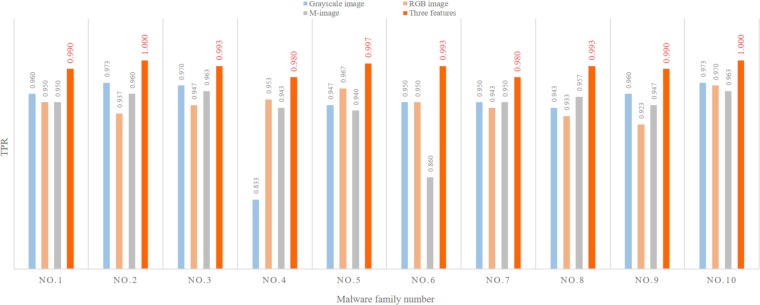
The influence of feature type and quantity on the result.

This section compares the influence of the number of features on the malware classification results. The results are shown in Fig 9. The ordinate in Fig 9 indicates the TPR, and the abscissa indicates the malware sample family number. The experimental results show that the multifeatured results are obviously better than when using only one feature when using the ELR integration strategy. Although using the ELR on one feature also achieves a good TPR, the classification may not be ideal for some malware families when only one feature view is adopted—for example, using only grayscale images to classify the No.4 family or using only M-images to classify the No.6 family. MHAS uses the multifeatured method to extract more comprehensive malware information, which helps to offset the overlap between some malware family classifications and improve the TPR. Fig 9 shows that when using the multifeature analysis method, MHAS can achieve a TPR of 100% for the 2nd and 10th families, while its lowest TPR is 98% for the 4th family.

#### Comparison of the results of MHAS and other analysis methods

**Fig 10:**
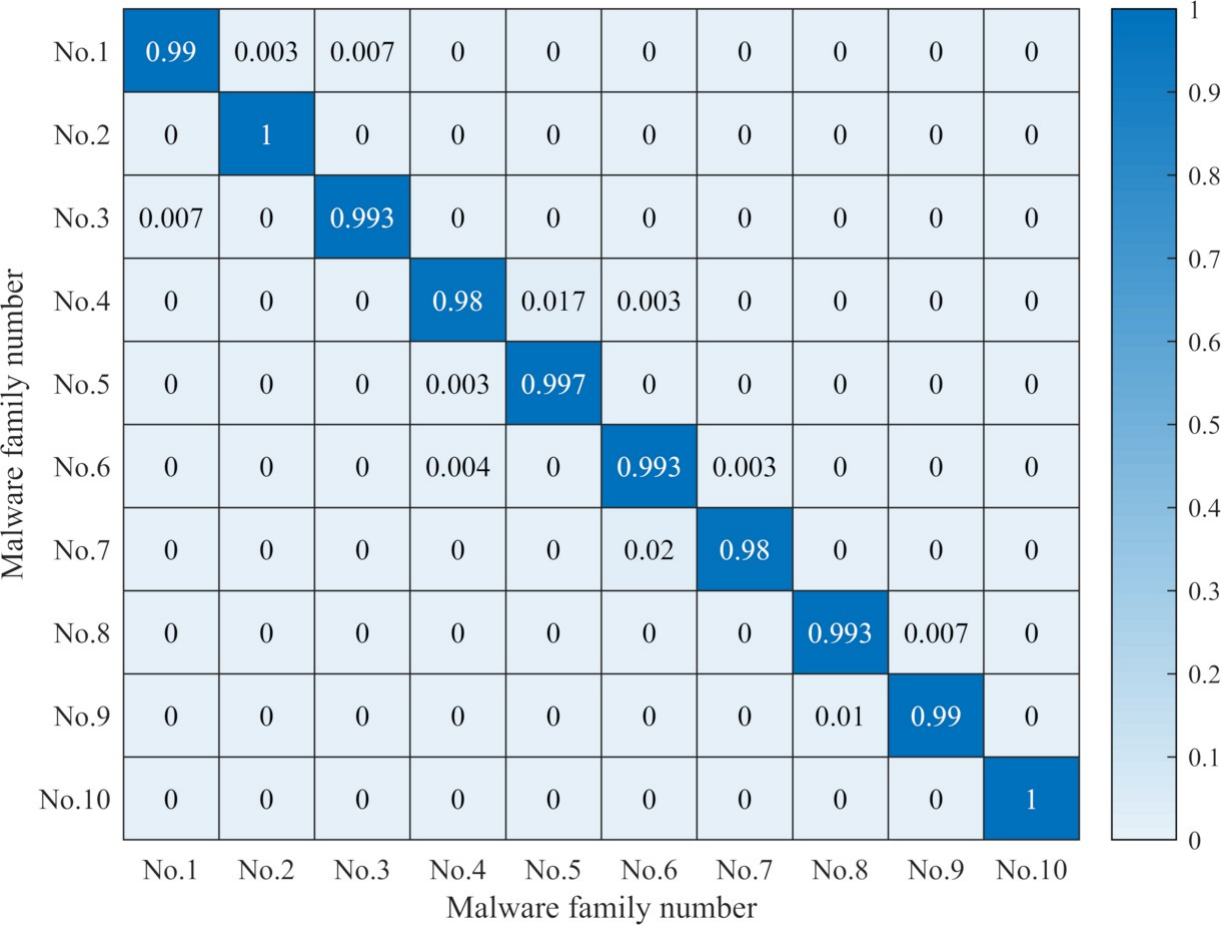
Confusion Matrix of malware family classification by the MHAS. The confusion matrix values are composed of the true positive rate and the false negative rate of the malware family classification by MHAS. The value of the subdiagonal represents the true positive rate, and the other values indicate the false negative rate. The true positive rate and false negative rate are the average values after 10-fold cross-validation.

For the 10 malware families, MHAS conducts the ELR of the three features, resulting in a confusion matrix as shown in Fig 10, where the ordinate and abscissa are the number of the malware family. The abscissa indicates the real malware family and the ordinate indicates the predicted malware family. The color patches in the figure indicate the similarity between the unknown sample and the known sample family. According to the ribbon on the right, the closer the color is to the top, the higher the similarity is, and the closer it is to the bottom, the lower the similarity is. Fig 10 shows that there is a small probability of false positives (confusions between the 4th family and the 6th family) belonging to the other family series. An analysis of the feature maps of both malware families shows that they have a small number of identical opcode sequences and system call subgraphs. As shown in Fig 10, MHAS has two characteristics: (1) the similarity between different malware families in the same series is higher than the similarity between different family series. Thus, even when a false alarm occurs, the predicted family is likely to belong to the same family series (e.g., the Backdoor series and the Trojan-Downloader series). (2) MHAS achieves a good performance regarding malware family classification. The unknown samples in each family have a high average similarity with the families in the signature database generated by the multifeature processing of known samples, while their average similarity with other families is lower.

**Fig 11:**
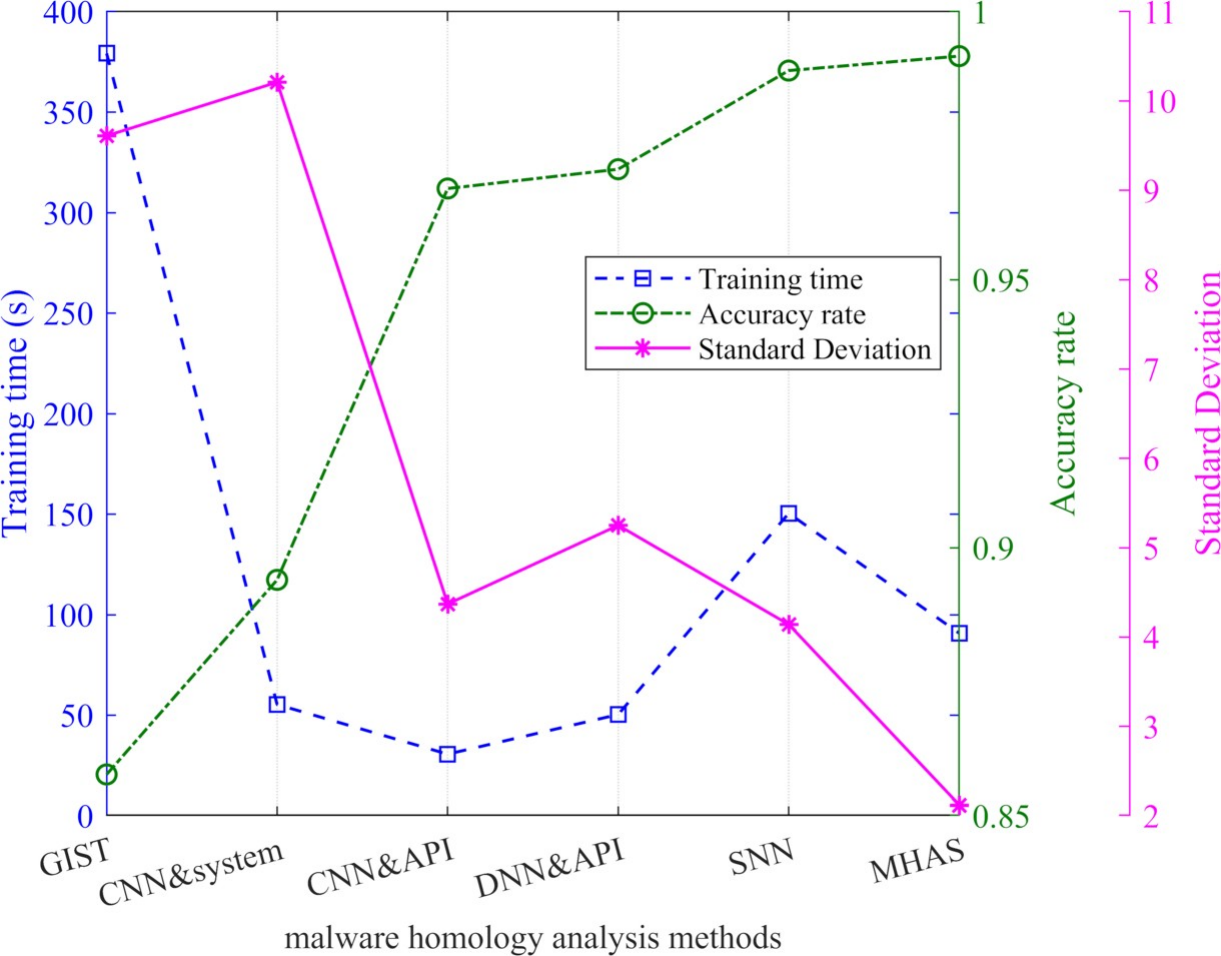
The results of six malware homology analysis methods.

The accuracy rate of malware classification is the key to identifying malware detection methods. By extracting multifeature information, MHAS learns and analyzes the base learner constructed from CNNs and the ELR. Finally, the AR of malware classification reaches 99.17%. Table 3 lists the accuracy rates of other malware homology analysis methods, including the GIST processing grayscale image texture fingerprints[17], a CNN processing system call sequences[21] and API call sequences[22], multitask learning and DNN processing API sequences[25], and an SNN processing grayscale images and opcode sequences[23]. From the data in Table 3, we can conclude that MHAS achieves a good AR in the field of malware homology analysis. In Fig 11 and Table 3, the MHAS is both faster and more accurate than GIST and SNN even though it processes both grayscale images and opcode sequences during training. Compared with the CNN, which processes system call sequences or API sequences and the DNN, which processes API sequences, MHAS improves the accuracy rate. Compared with the other five methods, MHAS achieves the smallest standard deviation, indicating that MHAS is more versatile for malware family classification and is suitable for classifying more malware families.

**Table 3:** The results of the different malware homology analysis methods.

Analytical method	Training time (s)	Accuracy rate	Standard Deviation
GIST	379.265	85.77%	9.61
CNN & system call sequences	55.349	89.4%	10.21
CNN & API sequences	30.632	96.7%	4.37
DNN & API sequences	50.343	97.06%	5.25
SNN & grayscale images and opcode sequences	150.447	98.9%	4.14
**MHAS**	**90.793**	**99.17%**	**2.12**

Table 3 shows that the accuracy rate of MHAS is only 0.27% higher than that of the SNN; consequently, we applied the Wilcoxon signed rank test [39] to these methods. When using MHAS and SNN to classify 10 malware families, the values of W^+^ and W^-^ are +21 and -7, respectively. For the bilateral test at alpha = 0.05, when n = 10, T^0.025^ = 8 by querying the distribution table of Wilcoxon signed rank test. Because W^+^>T^0.025^, H_0_ is accepted: there is no significant difference in the classification results of the two methods.

## Conclusions

This paper proposes a method based on ensemble learning and multifeatures views and constructs the MHAS system to address the problem that insufficient features are extracted during the process of malware homology analysis. First, MHAS extracts feature views consisting of grayscale images that represent binary information, RGB images that represent opcode sequences, and M-images that represent system call graphs. Second, to better study the three feature views, MHAS uses CNNs, which have good effects in image processing fields, as the base learners. Finally, to learning the results of the base learners, we propose the ELR method to improve the accuracy of malware analysis. MHAS mainly starts with static features, obtains the similarity measures of different malware through file outlines, instruction sequences, and control processes, performs homology analysis, and converges the results into different malware families. Moreover, MHAS can play an important role in tracking the origin of malware, investigations of the forensics and analysis of attack behaviors, attack method identification, and in deploying corresponding defense measures.

The experimental results show that MHAS can effectively analyze and identify malware families using static analysis methods, but the increasing complexity of malware has introduced additional confusion to static analysis methods. Therefore, in the next step, we will also consider operating system state changes before and after malware execution and use a combination of dynamic and static analysis to enrich the signature database and further improve the classification accuracy of malware families.

## Supporting information

S1 Dataset Malware samples dataset. The experimental dataset for this paper was collected primarily through the VX Heaven website, which contains 270,000 tagged malware samples.
(RAR)
