# Correction: Homology analysis of malware based on ensemble learning and multifeatures

**DOI:** 10.1371/journal.pone.0223679

**Published:** 2019-10-03

**Authors:** Di Xue, Jingmei Li, Weifei Wu, Qiao Tian, JiaXiang Wang

The first author, Di Xue, is incorrectly noted as the corresponding author. The correct corresponding author is Jingmei Li. Dr. Li’s email address is: lijingmei@hrbeu.edu.cn. Dr. Li’s ORCID iD is: 0000-0002-7246-3093 (https://orcid.org/0000-0002-7246-3093).
